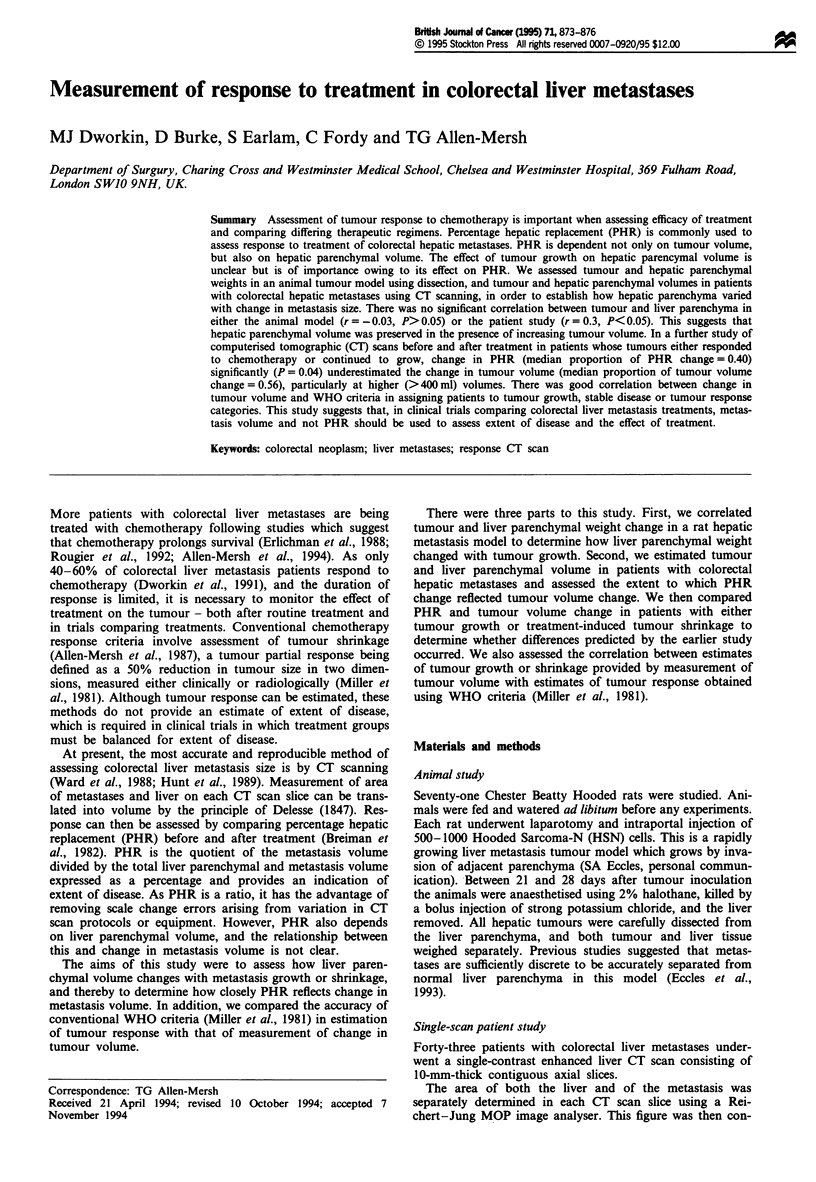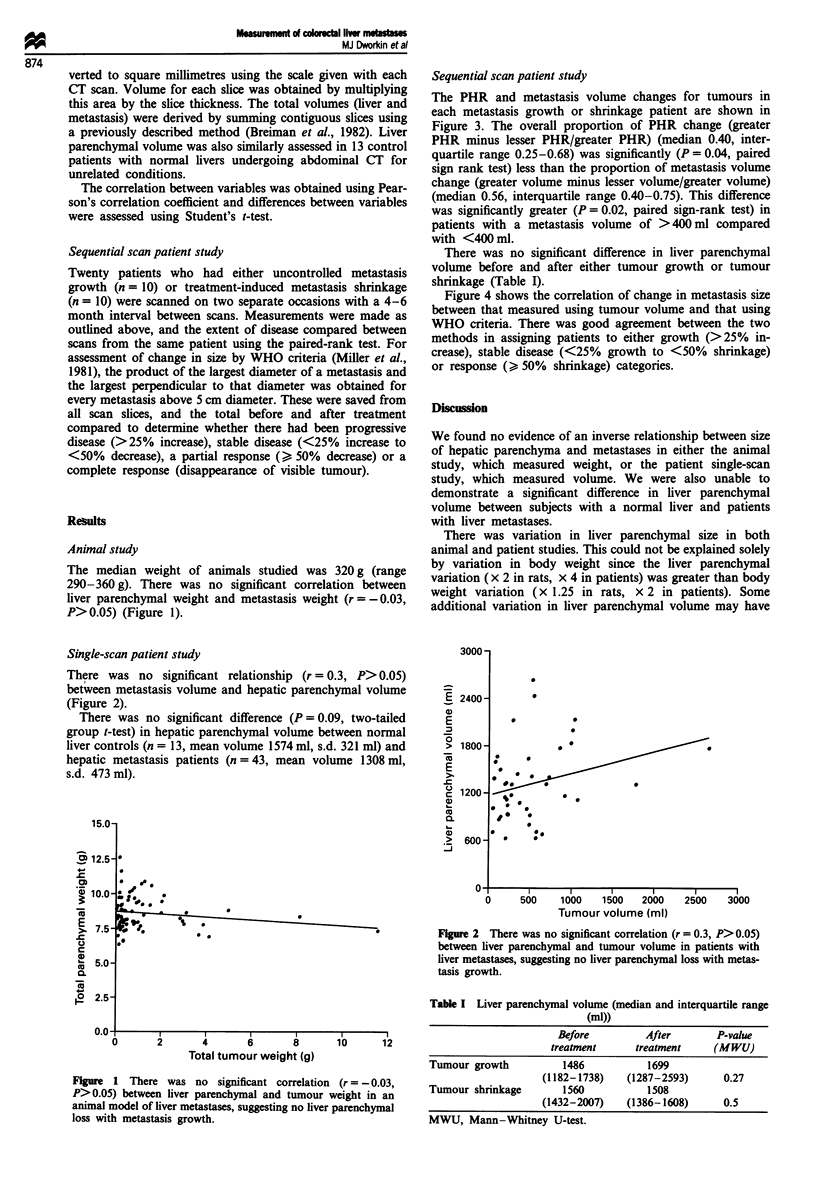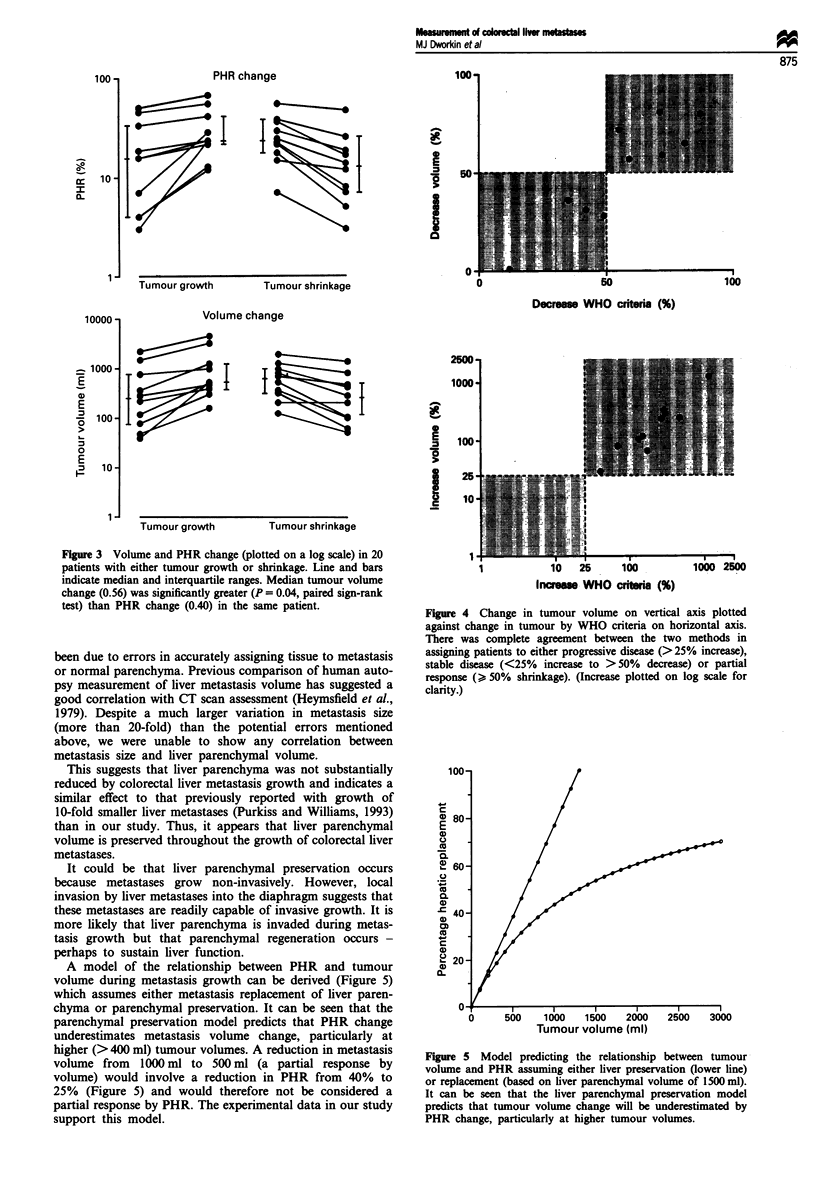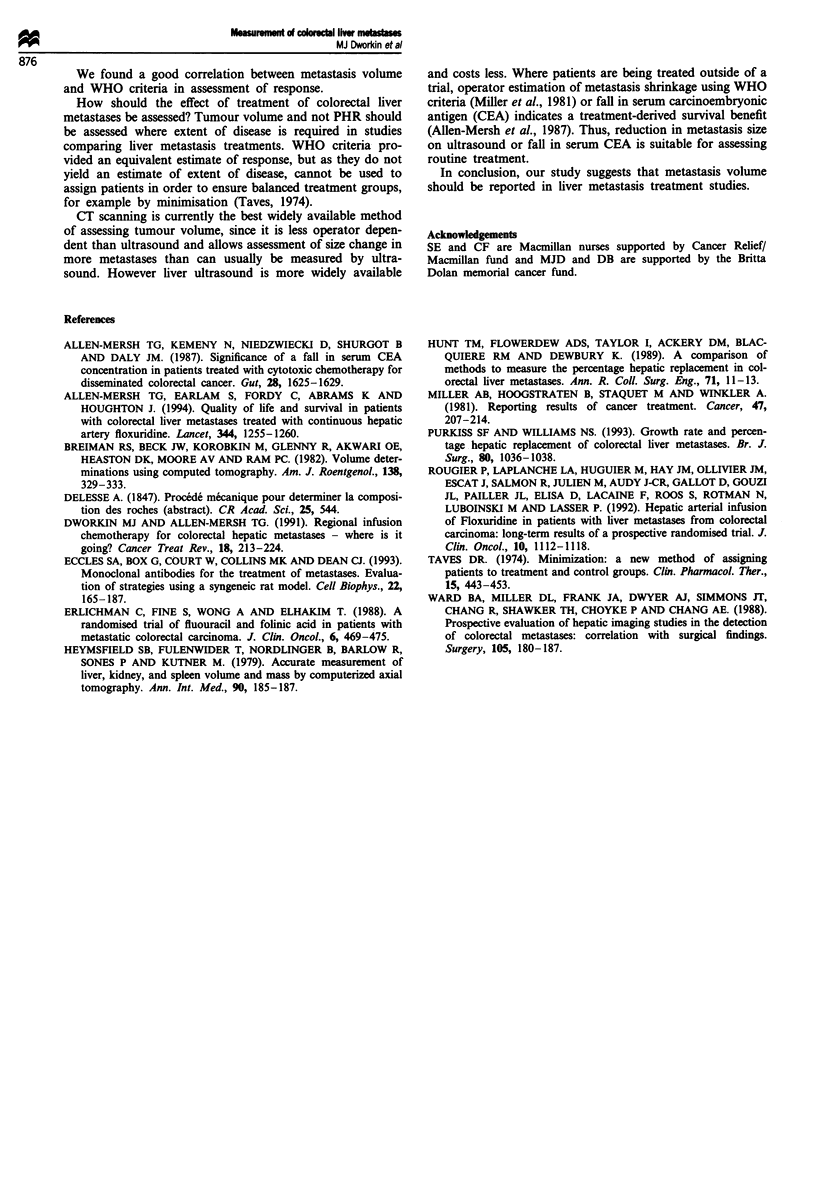# Measurement of response to treatment in colorectal liver metastases.

**DOI:** 10.1038/bjc.1995.168

**Published:** 1995-04

**Authors:** M. J. Dworkin, D. Burke, S. Earlam, C. Fordy, T. G. Allen-Mersh

**Affiliations:** Department of Surgery, Charing Cross and Westminster Medical School, Chelsea and Westminster Hospital, London, UK.

## Abstract

Assessment of tumour response to chemotherapy is important when assessing efficacy of treatment and comparing differing therapeutic regimens. Percentage hepatic replacement (PHR) is commonly used to assess response to treatment of colorectal hepatic metastases. PHR is dependent not only on tumour volume, but also on hepatic parenchymal volume. The effect of tumour growth on hepatic parenchymal volume is unclear but is of importance owing to its effect on PHR. We assessed tumour and hepatic parenchymal weights in an animal tumour model using dissection, and tumour and hepatic parenchymal volumes in patients with colorectal hepatic metastases using CT scanning, in order to establish how hepatic parenchyma varied with change in metastasis size. There was no significant correlation between tumour and liver parenchyma in either the animal model (r = -0.03, P > 0.05) or the patient study (r = 0.3, P < 0.05). This suggests that hepatic parenchymal volume was preserved in the presence of increasing tumour volume. In a further study of computerised tomographic (CT) scans before and after treatment in patients whose tumours either responded to chemotherapy or continued to grow, change in PHR (median proportion of PHR change = 0.40) significantly (P = 0.04) underestimated the change in tumour volume (median proportion of tumour volume change = 0.56), particularly at higher (> 400 ml) volumes. There was good correlation between change in tumour volume and WHO criteria in assigning patients to tumour growth, stable disease or tumour response categories. This study suggests that, in clinical trials comparing colorectal liver metastasis treatments, metastasis volume and not PHR should be used to assess extent of disease and the effect of treatment.


British Journal of Cancer (1995) 71, 873-876

© 1995 Stockton Press All rights reserved 0007-0920/95 $12.00

### Measurement of response to treatment in colorectal liver metastases

MJ Dworkin, D Burke, S Earlam, C Fordy and TG Allen-Mersh

*Department of Surgury, Charing Cross and Westminster Medical School, Chelsea and Westminster Hospital, 369 Fulham Road, London SW10 9NH, UK.*

**Summary** Assessment of tumour response to chemotherapy is important when assessing efficacy of treatment and comparing differing therapeutic regimens. Percentage hepatic replacement (PHR) is commonly used to assess response to treatment of colorectal hepatic metastases. PHR is dependent not only on tumour volume, but also on hepatic parenchymal volume. The effect of tumour growth on hepatic parencymal volume is unclear but is of importance owing to its effect on PHR. We assessed tumour and hepatic parenchymal weights in an animal tumour model using dissection, and tumour and hepatic parenchymal volumes in patients with colorectal hepatic metastases using CT scanning, in order to establish how hepatic parenchyma varied with change in metastasis size. There was no significant correlation between tumour and liver parenchyma in either the animal model ($r = -0.03$, $P > 0.05$) or the patient study ($r = 0.3$, $P < 0.05$). This suggests that hepatic parenchymal volume was preserved in the presence of increasing tumour volume. In a further study of computerised tomographic (CT) scans before and after treatment in patients whose tumours either responded to chemotherapy or continued to grow, change in PHR (median proportion of PHR change = 0.40) significantly ($P = 0.04$) underestimated the change in tumour volume (median proportion of tumour volume change = 0.56), particularly at higher (>400 ml) volumes. There was good correlation between change in tumour volume and WHO criteria in assigning patients to tumour growth, stable disease or tumour response categories. This study suggests that, in clinical trials comparing colorectal liver metastasis treatments, metastasis volume and not PHR should be used to assess extent of disease and the effect of treatment.

**Keywords:** colorectal neoplasm; liver metastases; response CT scan

More patients with colorectal liver metastases are being treated with chemotherapy following studies which suggest that chemotherapy prolongs survival (Erlichman *et al.*, 1988; Rougier *et al.*, 1992; Allen-Mersh *et al.*, 1994). As only 40-60% of colorectal liver metastasis patients respond to chemotherapy (Dworkin *et al.*, 1991), and the duration of response is limited, it is necessary to monitor the effect of treatment on the tumour - both after routine treatment and in trials comparing treatments. Conventional chemotherapy response criteria involve assessment of tumour shrinkage (Allen-Mersh *et al.*, 1987), a tumour partial response being defined as a 50% reduction in tumour size in two dimensions, measured either clinically or radiologically (Miller *et al.*, 1981). Although tumour response can be estimated, these methods do not provide an estimate of extent of disease, which is required in clinical trials in which treatment groups must be balanced for extent of disease.

At present, the most accurate and reproducible method of assessing colorectal liver metastasis size is by CT scanning (Ward *et al.*, 1988; Hunt *et al.*, 1989). Measurement of area of metastases and liver on each CT scan slice can be translated into volume by the principle of Delesse (1847). Response can then be assessed by comparing percentage hepatic replacement (PHR) before and after treatment (Breiman *et al.*, 1982). PHR is the quotient of the metastasis volume divided by the total liver parenchymal and metastasis volume expressed as a percentage and provides an indication of extent of disease. As PHR is a ratio, it has the advantage of removing scale change errors arising from variation in CT scan protocols or equipment. However, PHR also depends on liver parenchymal volume, and the relationship between this and change in metastasis volume is not clear.

The aims of this study were to assess how liver parenchymal volume changes with metastasis growth or shrinkage, and thereby to determine how closely PHR reflects change in metastasis volume. In addition, we compared the accuracy of conventional WHO criteria (Miller *et al.*, 1981) in estimation of tumour response with that of measurement of change in tumour volume.

Correspondence: TG Allen-Mersh
Received 21 April 1994; revised 10 October 1994; accepted 7 November 1994

There were three parts to this study. First, we correlated tumour and liver parenchymal weight change in a rat hepatic metastasis model to determine how liver parenchymal weight changed with tumour growth. Second, we estimated tumour and liver parenchymal volume in patients with colorectal hepatic metastases and assessed the extent to which PHR change reflected tumour volume change. We then compared PHR and tumour volume change in patients with either tumour growth or treatment-induced tumour shrinkage to determine whether differences predicted by the earlier study occurred. We also assessed the correlation between estimates of tumour growth or shrinkage provided by measurement of tumour volume with estimates of tumour response obtained using WHO criteria (Miller *et al.*, 1981).

### Materials and methods

### *Animal study*

Seventy-one Chester Beatty Hooded rats were studied. Animals were fed and watered *ad libitum* before any experiments. Each rat underwent laparotomy and intraportal injection of 500-1000 Hooded Sarcoma-N (HSN) cells. This is a rapidly growing liver metastasis tumour model which grows by invasion of adjacent parenchyma (SA Eccles, personal communication). Between 21 and 28 days after tumour inoculation the animals were anaesthetised using 2% halothane, killed by a bolus injection of strong potassium chloride, and the liver removed. All hepatic tumours were carefully dissected from the liver parenchyma, and both tumour and liver tissue weighed separately. Previous studies suggested that metastases are sufficiently discrete to be accurately separated from normal liver parenchyma in this model (Eccles *et al.*, 1993).

### *Single-scan patient study*

Forty-three patients with colorectal liver metastases underwent a single-contrast enhanced liver CT scan consisting of 10-mm-thick contiguous axial slices.

The area of both the liver and of the metastasis was separately determined in each CT scan slice using a Reichert-Jung MOP image analyser. This figure was then converted to square millimetres using the scale given with each CT scan. Volume for each slice was obtained by multiplying this area by the slice thickness. The total volumes (liver and metastasis) were derived by summing contiguous slices using a previously described method (Breiman *et al.*, 1982). Liver parenchymal volume was also similarly assessed in 13 control patients with normal livers undergoing abdominal CT for unrelated conditions.

The correlation between variables was obtained using Pearson's correlation coefficient and differences between variables were assessed using Student's *t*-test.

*Sequential scan patient study*

Twenty patients who had either uncontrolled metastasis growth ($n = 10$) or treatment-induced metastasis shrinkage ($n = 10$) were scanned on two separate occasions with a 4–6 month interval between scans. Measurements were made as outlined above, and the extent of disease compared between scans from the same patient using the paired-rank test. For assessment of change in size by WHO criteria (Miller *et al.*, 1981), the product of the largest diameter of a metastasis and the largest perpendicular to that diameter was obtained for every metastasis above 5 cm diameter. These were saved from all scan slices, and the total before and after treatment compared to determine whether there had been progressive disease (>25% increase), stable disease (<25% increase to <50% decrease), a partial response (⩾50% decrease) or a complete response (disappearance of visible tumour).

### Results

*Animal study*

The median weight of animals studied was 320 g (range 290–360 g). There was no significant correlation between liver parenchymal weight and metastasis weight ($r = -0.03$, $P > 0.05$) (Figure 1).

*Single-scan patient study*

There was no significant relationship ($r = 0.3$, $P > 0.05$) between metastasis volume and hepatic parenchymal volume (Figure 2).

There was no significant difference ($P = 0.09$, two-tailed group *t*-test) in hepatic parenchymal volume between normal liver controls ($n = 13$, mean volume 1574 ml, s.d. 321 ml) and hepatic metastasis patients ($n = 43$, mean volume 1308 ml, s.d. 473 ml).

**Figure 1** There was no significant correlation ($r = -0.03$, $P > 0.05$) between liver parenchymal and tumour weight in an animal model of liver metastases, suggesting no liver parenchymal loss with metastasis growth.

*Sequential scan patient study*

The PHR and metastasis volume changes for tumours in each metastasis growth or shrinkage patient are shown in Figure 3. The overall proportion of PHR change (greater PHR minus lesser PHR/greater PHR) (median 0.40, interquartile range 0.25–0.68) was significantly ($P = 0.04$, paired sign rank test) less than the proportion of metastasis volume change (greater volume minus lesser volume/greater volume) (median 0.56, interquartile range 0.40–0.75). This difference was significantly greater ($P = 0.02$, paired sign-rank test) in patients with a metastasis volume of >400 ml compared with <400 ml.

There was no significant difference in liver parenchymal volume before and after either tumour growth or tumour shrinkage (Table I).

Figure 4 shows the correlation of change in metastasis size between that measured using tumour volume and that using WHO criteria. There was good agreement between the two methods in assigning patients to either growth (>25% increase), stable disease (<25% growth to <50% shrinkage) or response (⩾50% shrinkage) categories.

### Discussion

We found no evidence of an inverse relationship between size of hepatic parenchyma and metastases in either the animal study, which measured weight, or the patient single-scan study, which measured volume. We were also unable to demonstrate a significant difference in liver parenchymal volume between subjects with a normal liver and patients with liver metastases.

There was variation in liver parenchymal size in both animal and patient studies. This could not be explained solely by variation in body weight since the liver parenchymal variation (×2 in rats, ×4 in patients) was greater than body weight variation (×1.25 in rats, ×2 in patients). Some additional variation in liver parenchymal volume may have

**Figure 2** There was no significant correlation ($r = 0.3$, $P > 0.05$) between liver parenchymal and tumour volume in patients with liver metastases, suggesting no liver parenchymal loss with metastasis growth.

**Table I** Liver parenchymal volume (median and interquartile range (ml))

| | *Before treatment* | *After treatment* | *P-value (MWU)* |
|---|---|---|---|
| Tumour growth | 1486 (1182–1738) | 1699 (1287–2593) | 0.27 |
| Tumour shrinkage | 1560 (1432–2007) | 1508 (1386–1608) | 0.5 |

MWU, Mann–Whitney U-test.

**Figure 3** Volume and PHR change (plotted on a log scale) in 20 patients with either tumour growth or shrinkage. Line and bars indicate median and interquartile ranges. Median tumour volume change (0.56) was significantly greater ($P = 0.04$, paired sign-rank test) than PHR change (0.40) in the same patient.

**Figure 4** Change in tumour volume on vertical axis plotted against change in tumour by WHO criteria on horizontal axis. There was complete agreement between the two methods in assigning patients to either progressive disease (>25% increase), stable disease (<25% increase to >50% decrease) or partial response (≥50% shrinkage). (Increase plotted on log scale for clarity.)

been due to errors in accurately assigning tissue to metastasis or normal parenchyma. Previous comparison of human autopsy measurement of liver metastasis volume has suggested a good correlation with CT scan assessment (Heymsfield *et al.*, 1979). Despite a much larger variation in metastasis size (more than 20-fold) than the potential errors mentioned above, we were unable to show any correlation between metastasis size and liver parenchymal volume.

This suggests that liver parenchyma was not substantially reduced by colorectal liver metastasis growth and indicates a similar effect to that previously reported with growth of 10-fold smaller liver metastases (Purkiss and Williams, 1993) than in our study. Thus, it appears that liver parenchymal volume is preserved throughout the growth of colorectal liver metastases.

It could be that liver parenchymal preservation occurs because metastases grow non-invasively. However, local invasion by liver metastases into the diaphragm suggests that these metastases are readily capable of invasive growth. It is more likely that liver parenchyma is invaded during metastasis growth but that parenchymal regeneration occurs – perhaps to sustain liver function.

A model of the relationship between PHR and tumour volume during metastasis growth can be derived (Figure 5) which assumes either metastasis replacement of liver parenchyma or parenchymal preservation. It can be seen that the parenchymal preservation model predicts that PHR change underestimates metastasis volume change, particularly at higher (>400 ml) tumour volumes. A reduction in metastasis volume from 1000 ml to 500 ml (a partial response by volume) would involve a reduction in PHR from 40% to 25% (Figure 5) and would therefore not be considered a partial response by PHR. The experimental data in our study support this model.

**Figure 5** Model predicting the relationship between tumour volume and PHR assuming either liver preservation (lower line) or replacement (based on liver parenchymal volume of 1500 ml). It can be seen that the liver parenchymal preservation model predicts that tumour volume change will be underestimated by PHR change, particularly at higher tumour volumes.

We found a good correlation between metastasis volume and WHO criteria in assessment of response.

How should the effect of treatment of colorectal liver metastases be assessed? Tumour volume and not PHR should be assessed where extent of disease is required in studies comparing liver metastasis treatments. WHO criteria provided an equivalent estimate of response, but as they do not yield an estimate of extent of disease, cannot be used to assign patients in order to ensure balanced treatment groups, for example by minimisation (Taves, 1974).

CT scanning is currently the best widely available method of assessing tumour volume, since it is less operator dependent than ultrasound and allows assessment of size change in more metastases than can usually be measured by ultrasound. However liver ultrasound is more widely available and costs less. Where patients are being treated outside of a trial, operator estimation of metastasis shrinkage using WHO criteria (Miller *et al.*, 1981) or fall in serum carcinoembryonic antigen (CEA) indicates a treatment-derived survival benefit (Allen-Mersh *et al.*, 1987). Thus, reduction in metastasis size on ultrasound or fall in serum CEA is suitable for assessing routine treatment.

In conclusion, our study suggests that metastasis volume should be reported in liver metastasis treatment studies.

**Acknowledgements**

SE and CF are Macmillan nurses supported by Cancer Relief/Macmillan fund and MJD and DB are supported by the Britta Dolan memorial cancer fund.

### References

ALLEN-MERSH TG, KEMENY N, NIEDZWIECKI D, SHURGOT B AND DALY JM. (1987). Significance of a fall in serum CEA concentration in patients treated with cytotoxic chemotherapy for disseminated colorectal cancer. *Gut*, **28**, 1625–1629.

ALLEN-MERSH TG, EARLAM S, FORDY C, ABRAMS K AND HOUGHTON J. (1994). Quality of life and survival in patients with colorectal liver metastases treated with continuous hepatic artery floxuridine. *Lancet*, **344**, 1255–1260.

BREIMAN RS, BECK JW, KOROBKIN M, GLENNY R, AKWARI OE, HEASTON DK, MOORE AV AND RAM PC. (1982). Volume determinations using computed tomography. *Am. J. Roentgenol.*, **138**, 329–333.

DELESSE A. (1847). Procédé mécanique pour determiner la composition des roches (abstract). *CR Acad. Sci.*, **25**, 544.

DWORKIN MJ AND ALLEN-MERSH TG. (1991). Regional infusion chemotherapy for colorectal hepatic metastases – where is it going? *Cancer Treat Rev.*, **18**, 213–224.

ECCLES SA, BOX G, COURT W, COLLINS MK AND DEAN CJ. (1993). Monoclonal antibodies for the treatment of metastases. Evaluation of strategies using a syngeneic rat model. *Cell Biophys.*, **22**, 165–187.

ERLICHMAN C, FINE S, WONG A AND ELHAKIM T. (1988). A randomised trial of fluouracil and folinic acid in patients with metastatic colorectal carcinoma. *J. Clin. Oncol.*, **6**, 469–475.

HEYMSFIELD SB, FULENWIDER T, NORDLINGER B, BARLOW R, SONES P AND KUTNER M. (1979). Accurate measurement of liver, kidney, and spleen volume and mass by computerized axial tomography. *Ann. Int. Med.*, **90**, 185–187.

HUNT TM, FLOWERDEW ADS, TAYLOR I, ACKERY DM, BLACQUIERE RM AND DEWBURY K. (1989). A comparison of methods to measure the percentage hepatic replacement in colorectal liver metastases. *Ann. R. Coll. Surg. Eng.*, **71**, 11–13.

MILLER AB, HOOGSTRATEN B, STAQUET M AND WINKLER A. (1981). Reporting results of cancer treatment. *Cancer*, **47**, 207–214.

PURKISS SF AND WILLIAMS NS. (1993). Growth rate and percentage hepatic replacement of colorectal liver metastases. *Br. J. Surg.*, **80**, 1036–1038.

ROUGIER P, LAPLANCHE LA, HUGUIER M, HAY JM, OLLIVIER JM, ESCAT J, SALMON R, JULIEN M, AUDY J-CR, GALLOT D, GOUZI JL, PAILLER JL, ELISA D, LACAINE F, ROOS S, ROTMAN N, LUBOINSKI M AND LASSER P. (1992). Hepatic arterial infusion of Floxuridine in patients with liver metastases from colorectal carcinoma: long-term results of a prospective randomised trial. *J. Clin. Oncol.*, **10**, 1112–1118.

TAVES DR. (1974). Minimization: a new method of assigning patients to treatment and control groups. *Clin. Pharmacol. Ther.*, **15**, 443–453.

WARD BA, MILLER DL, FRANK JA, DWYER AJ, SIMMONS JT, CHANG R, SHAWKER TH, CHOYKE P AND CHANG AE. (1988). Prospective evaluation of hepatic imaging studies in the detection of colorectal metastases: correlation with surgical findings. *Surgery*, **105**, 180–187.